# Effect of Supplementation Plans and Frequency on Performance and Metabolic Responses of Grazing Pregnant Beef Heifers

**DOI:** 10.3390/vetsci11100506

**Published:** 2024-10-15

**Authors:** Luciano Prímola de Melo, Luciana Navajas Rennó, Edenio Detmann, Mário Fonseca Paulino, Ronaldo Gomes da Silva Júnior, Román Maza Ortega, Deilen Sotelo Moreno

**Affiliations:** 1 Animal Science Department, Federal University of Viçosa, Viçosa 36570-900, MG, Brazil; lucianoprimola@gmail.com (L.P.d.M.); lucianarenno@ufv.br (L.N.R.); detmann@ufv.br (E.D.); mariofonsecapaulino@gmail.com (M.F.P.); ronaldo.g.junior@ufv.br (R.G.d.S.J.); 2 Animal Science Department, Faculty of Agricultural Sciences, University of Pamplona, Pamplona 543050, NDS, Colombia; roman.maza@unipamplona.edu.co; 3 Animal Production Department, Faculty of Agricultural and Animal Sciences and Natural Resources, University of the Llanos, Villavicencio 500017, MET, Colombia

**Keywords:** carry-over, Nellore, primiparous, super-precocious, tropical pasture

## Abstract

**Simple Summary:**

Protein supplementation combined with infrequent supplementation strategies for grazing cattle aims to correct dietary and metabolic deficiencies of tropical grasses, optimize animal performance, and reduce costs in grazing cattle production systems. However, many studies in tropical conditions have only focused on evaluating the effects of daily supplementation in beef cattle. This study aimed to evaluate the effects of both frequency and plan of protein supplementation for super-precocious pregnant Nellore heifers under grazing. We observed that the frequency or supplementation plan did not influence the intake of total dry matter and forage. However, daily and infrequent supplementation increased the crude protein and organic matter intake, which led to a higher average daily gain, body weight at calving, and subcutaneous thickness fat during the prepartum period, as well as higher serum urea nitrogen and insulin-like growth factor-I and lower non-esterified fatty acid concentrations. In contrast, β-hydroxybutyrate and progesterone blood levels were not affected by daily or infrequent supplementation or supplementation plans. Our results showed that infrequent supplementation during beef heifers’ gestation does not harm their productive performance or metabolic status.

**Abstract:**

Our objective was to evaluate the effects of protein supplementation frequency and supplementation plans during gestation on productive performance and metabolic responses of grazing super-precocious Nellore heifers. Thirty-five pregnant Nellore heifers were used in a completely randomized design in a 2 × 2 factorial arrangement with an additional control group. The factors were the following: 1. Frequency of supplementation: (a) Daily (seven times per week), (b) Infrequent (three times per week); 2. Supplementation plans: (a) Constant, (b) Increasing. A control group with no supplementation was included. Two digestibility trials were carried out on the 40th and 130th experimental days. Productive performance and body composition were evaluated in the prepartum and postpartum periods. Blood samples were collected at −114, −113, −15, −14, +15, +30, and +45 days relative to calving for measuring metabolic status. Supplementation (daily and infrequent) increased the intake of total dry matter (DM) (*p* ≤ 0.004), average daily gain (*p* < 0.001), and body weight at calving (*p* = 0.008) at prepartum. However, frequency or supplementation plan did not alter (*p* > 0.17) the intake of total DM and forage DM. There was an effect (*p* ≤ 0.02) of the supplementation plan on subcutaneous thickness fat in the prepartum period and albumin and non-esterified fatty acid concentrations (*p* < 0.004) in the postpartum period. Nonetheless, frequency and supplementation plans did not affect (*p* ≥ 0.10) heifers’ productive performance during the postpartum period. In conclusion, protein supplementation frequency and supplementation plan during beef heifers’ gestation do not negatively impact their productive performance or metabolic responses.

## 1. Introduction

Beef cattle production systems in tropical regions typically are characterized by dietary deficiencies of tropical grasses and metabolic disorders of the cattle [1]. Among the factors affecting these parameters, nutrition possibly has the most significant impact [2]. This fact is more prominent in super-precocious primiparous dams, as they require an optimal plane of nutrition and a high average daily gain (ADG) to reach 55–60% of their mature body weight by 14 months of age. Additionally, their conception and calving occur at 14 and 23 months of age, respectively, necessitating adequate energy and protein for their continued growth [3]. Consequently, these mothers and their offspring are particularly affected by situations of nutritional imbalance.

In tropical areas, such as Brazil, the cattle breeding and peripartum season often coincides with the season of poor forage availability and quality, which may be inadequate to meet their nutritional requirements. As a result, at least in the absence of supplementation, grazing pregnant heifers must more intensively mobilize their body reserves of energy and protein [4] to guarantee their growth and supply of nutrients to the fetus, as forage alone is insufficient to meet these demands. Hence, supplementation strategies are often required to reduce these deficiencies and improve nutritional, productive, and metabolic performances of pregnant super-precocious heifers [5]. However, due to their high cost, it is necessary to adopt rational supplementation strategies, such as reducing the frequency of supplementation and adapting the amount offered to suit demand. These factors can make supplementation more efficient by reducing costs and maintaining performance. Thus, the decrease in the supplementation frequency is a strategy characterized by operational cost reduction and with physiological support in ruminants [6], possibly associated with the ability of ruminants to recycle a percentage of the nitrogen consumed to the rumen, thus allowing them to explore the carry-over effects of protein supplementation even on days without offering supplements [6,7,8].

Results obtained in previous research indicated that protein supplements could be offered every 3 to 10 days without negative effects on the efficient use of nitrogen and productive performance [8,9]. Other studies have provided evidence that protein supplementation can also reflect on their progeny, even if given infrequently, in addition to enhancing the productive and metabolic performance of the dams [9,10]. Nonetheless, it has also been reported that the infrequency of protein supplementation leads to an increase in protein intake per meal, as well as the size of each meal [11]. This excessive protein intake can increase serum urea nitrogen concentrations and reduce uterine pH, likewise altering the synthesis and release of hormones and metabolites associated with energy and protein metabolism [11,12,13,14,15]. Thus, supplementation may be particularly important when provided in the last third of gestation.

Several studies have evaluated the effects of prepartum supplementation on productive and metabolic responses in beef cows [9,16,17,18]. However, we are unaware of studies that have evaluated the effects of differing plans of a protein supplementation provided infrequently on metabolic and hormonal responses and productive performance of grazing super-precocious beef heifers during pregnancy consuming a low-quality pasture.

Therefore, we hypothesized that providing high nutritional supplementation during the beef heifers’ gestation improves their performance and metabolic characteristics at both prepartum and postpartum and that infrequent supplementation (three times a week) does not negatively influence their performance. Thereby, our objective was to evaluate the effects of supplementation plans and protein supplementation frequency during gestation on productive performance and metabolic responses of grazing super-precocious Nellore heifers.

## 2. Materials and Methods

### 2.1. Location and Weather Conditions

The experiment was conducted at the Beef Cattle Farm of the Animal Science Department of the Federal University of Viçosa, Viçosa-MG, Brazil (20°45′ S, 42°52′ W), between April and December 2017, corresponding to the rainy–dry, dry, and dry–rainy seasons (249 experimental days). The experimental area was located in a mountainous region with an altitude of 670 m. The average rainfall during the experiment was 74.9 mm (44.8, 51.4, 18.6, 1.8, 0.8, 14.0, 47.0, 106.0, and 389.8 mm), with an average temperature of 19.4 °C (20.5, 18.2, 17.7, 15.8, 17.1, 19.0, 22.4, 21.5, and 22.5 °C) and a medium relative humidity of 76.5% (81.4, 83.5, 82.2, 78.6, 74.1, 66.1, 66.2, 76.9, and 79.3%) for April, May, June, July, August, September, October, November, and December, respectively [19].

### 2.2. Animal Management, Experimental Design, and Treatments

Thirty-five pregnant Nellore heifers were used, averaging 22.4 ± 1.68 months old, a 407 ± 21.6 kg initial body weight (BW), and a 5.7 ± 0.20 body condition score (BCS; on a scale of 1 to 9). Heifers were pregnant at an average of 14 months old by a fixed-time artificial insemination (FTAI) protocol and inseminated by the same technician using semen doses of a bull of the Nellore breed. Two synchronization protocols were performed as follows: the first protocol: on day 0, an intravaginal device of progesterone release (Tecnopec Primer, São Paulo, Brazil) was introduced, and heifers received an injection of 2.0 mg of estradiol benzoate (Tecnopec Primer, São Paulo, Brazil). On day 8, the intravaginal device was removed, and a 2 mL injection of cloprostenol sodium (MSD Saúde Ciosin Animal, São Paulo, Brazil) was administered. On day 9, heifers received 0.5 mL of estradiol cypionate via injection (Zoetis-Pfizer E.C.P., Campinas, Brazil), and all were inseminated 30 h later. The second protocol: twenty days after FTAI, heifers were subjected to resynchronization using the previously described protocol. On day 28 after FTAI, pregnancy diagnosis was performed via transrectal ultrasound evaluation (Aloka SSD 500, 5 MHz linear transducer, Aloka Co., Tokyo, Japan). A pregnant female was considered to be one with the presence of amniotic fluid and an embryo with a heartbeat in the uterine lumen, and was excluded from the resynchronization protocol. On the same day, the intravaginal device was removed from all heifers and non-pregnant heifers received a 2 mL injection of cloprostenol sodium (MSD Saúde Ciosin Animal, São Paulo, Brazil). On day 29 after FTAI, non-pregnant heifers received 0.5 mL of estradiol cypionate via injection (Zoetis-Pfizer E.C.P., Campinas, Brazil) and all were inseminated again 30 h later. A final pregnancy diagnosis was conducted 60 days after the first and second FTAI.

All heifers were randomly allocated into ten 2 ha paddocks each (two paddocks for each treatment), evenly covered with Signal grass (*Urochloa decumbens*), and equipped with drinkers and feeders. The study started at 189-days prepartum until day 60 of lactation, corresponding to 249 experimental days.

The nomenclature for each animal was set at the beginning of the experiment and used throughout the manuscript, even though after calving, the category changed (e.g., heifers became primiparous cows). As the evaluations were focused on individual performance and these measurements were collected individually, the animal was considered the experimental unit (seven replicates by treatment), as recommended by Detmann et al. [20].

The experimental design was completely randomized in a 2 × 2 factorial arrangement with an additional control group. The factors were as follows: 1. Frequency of supplementation (2 levels): (a) Daily (seven times per week); (b) Infrequent (three times per week; Monday, Wednesday, Friday); 2. Supplementation plans (2 levels): (a) Constant (CO; 1.0 kg/day in the both the middle and last third of gestation); (b) Increasing (IN; 0.5 and 1.5 kg/day in the middle and last third of gestation, respectively). A control group with no supplementation was included for comparison, resulting in 5 treatments: Daily-CO, Daily-IN, Infrequent-CO, Infrequent-IN, and control treatment. All supplemented treatments received the same total amount of supplement throughout the experiment (189 kg per animal).

The supplement on an as-fed basis was composed of 975 g/kg wheat meal, 22.5 g/kg urea, and 2.5 g/kg ammonium sulfate and formulated to contain 250 g/kg crude protein (CP; Table 1) and fed daily at 1100 h in a collective feeder to minimize any interference with animal grazing behavior, which is experimental handling closer to what is observed in beef production systems due to cattle gregarious behavior. All animals had free access to water and a mineral mixture (500 g/kg CaHPO_4_; 471.9 g/kg NaCl; 15 g/kg ZnSO_4_; 7 g/kg Cu_2_SO_4_; 500 mg/kg CoSO_4_; 500 mg/kg KIO_3_; 100 mg/kg Na_2_SeO_3_, and 5 g/kg MnSO_4_).

The quantity of 1.0 kg/animal/day of protein supplement adopted for daily supplementation corresponds to approximately 25% of the CP dietary requirements of a pregnant Zebu beef heifer with a BW of 450 kg and an expected gain of 0.15 kg/day [21]. The other supplemented treatments were based on the provision of the same total amount of CP but offered infrequently and/or in increasing amounts.

### 2.3. Food and Feces Sampling and Chemical Analysis

Representative samples of supplements were collected monthly for chemical analysis. Forage chemical composition was determined by hand-plucked samples that were collected every 15 days based on the identification of the places of intake and the parts of the plant selected by the animals, simulating the heifers’ grazing as closely as possible [22] (Table 1). A second pasture sample was collected every 30 days, consisting of four forage subsamples randomly collected in each paddock by cutting approximately 1 cm above the ground using a metal square (0.5 m × 0.5 m). Samples were prepared in a forced air circulation oven and partially dried at 60 °C for 72 h and ground in a knife Willye mill (model 3; Arthur H. Thomas, Philadelphia, PA, USA) to pass through a 2 mm screen. After that, half of each ground sample was ground again to pass through a 1 mm screen.

To evaluate the intake and digestibility of nutrients in all heifers, two digestibility trials (lasting 12 days each) were carried out, the first from the 40th experimental day and the second from the 130th experimental day. Chromium oxide (Cr_2_O_3_) was utilized as an external marker to assess fecal excretion with a dosage of 20 g per animal [20]. The Cr_2_O_3_ was packed in paper cartridges and administered through the esophagus using a metal probe, once daily at 1100 h over 11 days. Individual intake of the supplement was estimated using titanium dioxide (TiO_2_) mixed into the supplement in a plastic bag in a proportion of 10 g/kg of supplement for 11 days [20]. Additionally, indigestible neutral detergent fiber (iNDF) served as an internal marker to estimate forage dry matter (DM) intake [20]. The first 5 days were designated for the stabilization of the excretion of the markers, and fecal samples were collected immediately after defecation or directly from the rectum of the animals in amounts of approximately 200 g at 1800, 1600, 1400, 1200, 1000, 0800, and 0600 h on days 6, 7, 8, 9, 10, 11, and 12 of the digestion trial, respectively. All the fecal samples were identified, partially oven-dried at 60 °C for 72 h, and ground as described for forage samples. Grounded samples were pooled over the sampling time points by heifers and stored in plastic pots before analysis.

Samples of supplements, forage, and feces ground to 2 mm were analyzed for iNDF (after 288 h of ruminal in situ incubation; INCT-CA F-009/1). Samples ground to 1 mm were analyzed DM (dried overnight at 105 °C; method INCT-CA G-03/1), ash (complete combustion in a muffle furnace at 600 °C for 4 h; method INCT-CA M-001/1), CP (Kjeldahl procedure; method INCT-CA N-001/1), ether extract (EE—Randall procedure; method INCT-CA G-005/1), and NDF corrected for ash and protein (apNDF: using a heat-stable α-amylase, omitting sodium sulfite, and correcting for residual ash and protein; methods INCT-CA F-002/1; INCT-CA M-002/1; INCT-CA N-004/1), according to the standard analytical procedures of the Brazilian National Institute of Science and Technology in Animal Science (INCT-CA) [23].

Feces samples were also analyzed for chromium concentration using nitroperchloric digestion and atomic absorption spectrophotometry (GBC Avanta Σ, Scientific Equipment, Braeside, Victoria, Australia; method INCT-CA M-005/1) according to Detmann et al. [24]. The fecal DM excretion was estimated using the Cr_2_O_3_ marker, based on the ratio between the amount of chromium supplied and its concentration in the feces, as recommended by Detmann et al. [20].

Fecal DM (kg/day) = AII/ICF

where AII = amount of indicator ingested (g) and ICF = indicator concentration in fecal DM (g/kg of fecal DM).

Individual supplement intake (ISI) was estimated by the ratio of excretion of TiO_2_ in feces and marker concentration in the supplement, as recommended by Detmann et al. [20]:

ISI (kg/day) = [(FE × ICaF)/IOG] × SOG

where FE = fecal DM excretion (kg/day); ICaF = indicator concentration in animal feces (kg/kg); IOG = indicator present in the supplement offered to each group (kg/day); and SOG = supplement amount offered to the group of animals or treatment (kg/day).

Individual DM intake (DMI) was estimated by using iNDF as an internal marker and calculated by the following equation described by Detmann et al. [20]:

DMI (kg/day) = [(FE × iNDFF − iNDFS)/iNDFP] + ISI

where iNDFF = concentration of iNDF in the feces (kg/kg); iNDFS = concentration of iNDF in the supplement (kg/kg); and iNDFP = amount of iNDF from pasture (kg).

Digested organic matter (DOM) was calculated according to Detmann et al. [21]:

DOM (kg) = (IOMS + IFOM) × DOOM

where IOMS = intake organic matter from the supplement; IFOM = intake forage organic matter; and DOOM = digestibility of organic matter.

### 2.4. Productive Performance

For productive performance evaluation, heifers were weighed at the beginning of the trial, 90 days prior to calving, weekly from 15 days before the expected date of calving (to obtain the BW estimated at calving; BWec), and 60 days postpartum. Calves were weighed at birth and at 60 days of age to evaluate their performance. Additionally, every 30 days, all heifers were weighed to monitor performance and animal welfare. All the BWs were obtained at 0600 h, except on the day of calving.

The BWec was calculated using the following equation:

BWec = BWbc + [(BWbc − BWblt)/(Dlw − Dblt)] × (Dac − Dlw)

where BWbc = body weight at the last week weighing before calving; BWblt = body weight at the beginning of the last third of gestation; Dlw = date of last weighing; Dblt = date of beginning of the last third of gestation; and Dac = day of actual calving.

Upon analysis, the BWs were corrected to shrunk BW (SBW) [24] in order to avoid the possible confounding effect of the last meal filling the digestive tract:

SBW (kg) = 0.8084 × BW^1.0303^

To evaluate the body composition (muscle and fat) of the animals at the beginning of the experiment, 15 days before the expected calving date, and at the end of the experiment, ribeye area (REA) and subcutaneous thickness fat (STF) over the longissimus dorsi (between the 12th and 13th ribs) and subcutaneous thickness fat over the biceps femoris muscle (between the ischium and pubis) were recorded with an ultrasound (Aloka SSD 500; 3.5 MHz linear probe; Aloka Co. Ltd., Wallingford, CT, USA). Images were analyzed in the BioSoft Toolbox II for Beef software (Biotronics Inc., Ames, IA, USA). The STF was estimated as the average of the values obtained in the region of the longissimus dorsi and biceps femoris muscle. Simultaneously, the BCS was recorded by three experienced technicians on a scale of 1 to 9, as recommended by the NASEM [25].

On days 35 and 50 postpartum (peak lactation), milk samples were collected to estimate the cows’ milk composition and production. Milking procedures were followed as described by Almeida et al. [26], which used a controlled suckling period before the calf separation. In order to deplete the milk produced by cows, calves were separated from dams at 1500 h; the cows returned to paddock while the calves remained in the cattle shed. At 1730 h, the calves were reunited with their dams and allowed to suckle for 30 min. At 1800 h, calves were once again separated from mothers until the next morning. At 0600 h on the next day, cows were milked mechanically immediately after an injection of 2 mL of oxytocin (10 IU/mL; Ocitovet^®^, Vet&Cia Animal Health, São Paulo, Brazil) in the mammary artery, and the produced milk was weighed immediately after milking. The exact time when the milking of each cow ended was recorded, and the milk yield was converted to a 24 h production.

Individual samples of 50 mL of milk were taken for analyses of protein, fat, lactose, and total solids. Samples were stored at 4 °C in a refrigerator using a bronopol tablet per sample as a preservative. Milk samples were analyzed using spectroscopy (Foss MilkoScan FT120, Hillerød, Denmark). Milk production was corrected to 4% of fat (Milk_4%_) according to the NRC [27]:

Milk_4%_ (kg) = 0.4 × (milk production) + [15 × (fat production × milk production/100)]

### 2.5. Blood Metabolite and Hormone Assessment

Blood samples were collected as a function of days in relation to calving as follows: −114 and −113 for the middle third of gestation; −15 and −14 for the last third of gestation, always coinciding with Wednesdays and Thursdays, comprising a day when both daily heifers and infrequent heifers were supplemented, and a day on which infrequent heifers did not receive a supplement, respectively; and +15, +30, and +45 days for the postpartum period for quantification of urea, total proteins, albumin, glucose, non-esterified fatty acids (NEFA), β-hydroxybutyrate (βHB), insulin-like growth factor-I (IGF-1), and progesterone concentrations. Blood samples were collected at 0700 h via puncture of the jugular vein using vacuum tubes with sodium fluoride (glycolytic inhibitor) and ethylenediaminetetraacetic acid (EDTA; anticoagulant; BD Vacutainer^®^ Fluoride/EDTA, BD, São Paulo, Brazil) for glucose analysis and tubes with vacuum with separating gel and coagulation activator (BD Vacutainer^®^ SST II Advance) for other analyses. Blood was kept at 4 °C and centrifuged (1450× *g* for 15 min), and serum and plasma were frozen at −20 °C immediately for later analysis.

Blood glucose (K082) and urea (K056) concentrations were quantified by enzymatic colorimetric methods. Total proteins (K031) and albumin (K040, Bioclin^®^ Quibasa, Belo Horizonte, Brazil) were analyzed by colorimetric methods in an automatic biochemistry analyzer (Mindray BS200E, Shenzhen, China). Blood NEFA (FA115) was analyzed by the colorimetric method, and βHB (RB1007, Randox^®^ Laboratories Ltd., Antrim, UK) was analyzed by the enzymatic method. Serum urea nitrogen (SUN) was estimated as 46.67% of the total serum urea. The metabolites were analyzed using an automatic biochemistry analyzer (Mindray BS200E, Shenzhen, China). The IGF-1 (313231, DiaSorin, Vercelli, Italy) and progesterone concentrations (33550, Beckman Coulter^®^, Brea, CA, USA) were analyzed by indirect chemiluminescence method in the Liaison analyzer (DiaSorin, Saluggia, Italy) and Access^®^ 2 Immunoassay System (Beckman Coulter Inc., Brea, CA, USA), respectively.

### 2.6. Statistical Analysis

Response variables were analyzed using the MIXED procedure in SAS 9.4 (SAS Institute Inc., Cary, NC, USA). Comparisons between treatments were performed according to a 2 × 2 factorial arrangement with an additional control group as follows: 1. Frequency of supplementation (2 levels): (a) Daily, (b) Infrequent; 2. Supplementation plans (2 levels): (a) Constant, (b) Increasing; 3. Control group with no supplementation, using orthogonal contrasts constructed in order to evaluate the effects of supplementation (control vs. daily and infrequent supplementation), frequency of supplementation (daily vs. infrequent), supplementation plans (constant vs. increasing), and their interactions (daily and infrequent vs. constant and increasing). For the variables that did not present a supplementation effect but a frequency of supplementation or supplementation plan effect was significant, a Dunnett’s test was performed to identify whether a supplemented treatment differed from the control.

The effect of treatment on all variables measured was evaluated by ANOVA, adopting the initial BW as the covariate, according to the following mathematical model:

Y_ijk_ = μ + α_i_ + β_j_ + (αβ)_ij_ + e_(i)j_ + ε_(ij)k_

where Y_ijk_ = observations of individual k on paddock j under treatment i; μ = overall mean; α_i_ = fixed effect of daily and infrequent supplementation; β_j_ = fixed effect of supplementation plan; (αβ)_ij_ = interaction effect between frequency and the supplementation plan; e_(i)j_ = random error, unobservable, associate to each j paddock under treatment i, assumed to be normally and independently distributed (NID; 0, σe^2^); and ε_(ij)k_: random error, unobservable, associate to each k observation on j paddock under treatment i, assumed to be NID (0, σe^2^).

The blood metabolites and hormones, and milk production and composition, were analyzed as repeated measurements over time. The Mauchly’s test of sphericity was not significant; therefore, the sphericity assumption is met. The choice of the best covariance matrix was performed following the Akaike information criteria with correction. The degrees of freedom were estimated according to the Kenward–Roger method. The data showed normality by the Shapiro–Wilk test and homoscedasticity through the Bartlett test. Differences were considered significant at *p* ≤ 0.05, and tendencies were considered at 0.05 ≤ *p* ≤ 0.10.

## 3. Results

### 3.1. Forage Samples and Nutritional Performance

The forage consumed by heifers in the experimental period presented an average DM for the experimental period of 3.16 t/ha. The pasture showed lower CP values in the prepartum period and higher during the postpartum period, whereas apNDF progressively decreased throughout the experiment periods (Table 1).

No frequency × supplementation plan interactions were detected on intake and total digestibility of diet components and performance of the heifers during the experiment. Additionally, no frequency (daily or infrequent supplementation) effects (*p* > 0.10) were observed in the experiment; therefore, only the main effects are discussed separately.

Supplementation increased (*p* < 0.05) the intake of organic matter (OM; average of 6.16 vs. 4.08 kg/day) and CP (average of 0.54 vs. 0.22 kg/day), while the intake of total DM (average of 6.58 vs. 4.62 kg/day; *p* = 0.062), apNDF (average of 4.27 vs. 3.10 kg/day; *p* = 0.059), and iNDF (average of 1.5 vs. 1.19 kg/day; *p* = 0.095) tended to be greater for supplemented (daily and infrequent) heifers compared to control heifers in the middle third of gestation (Table 2). In the middle third of gestation, supplementation did not affect (*p* > 0.10) the intake of forage DM, DOM, or CP/DOM ratio. Frequency or supplementation plan did not alter (*p* > 0.10) the intake of the diet components, except for CP intake, which tended to be greater in CO heifers compared to IN heifers (average of 0.63 vs. 0.45 kg/day, respectively).

In the last third of gestation, there was a greater (*p* < 0.05) intake of total DM (average of 5.87 vs. 4.47 kg/day), OM (average of 5.53 vs. 4.18 kg/day), CP (average of 0.55 vs. 0.22 kg/day), apNDF (average of 3.64 vs. 3.09 kg/day), DOM (average of 2.33 vs. 1.56 kg/day), and CP/DOM ratio for supplemented (average of 232 vs. 139 g/kg) heifers compared to control heifers (Table 2). However, supplementation did not affect (*p* > 0.10) forage DM and iNDF intake. Likewise, frequency or supplementation plan did not alter (*p* > 0.10) the intake of total DM, forage DM, OM, apNDF, iNDF, DOM, and CP/DOM ratio. Nonetheless, the supplementation plan affected CP intake, being greater in IN heifers compared to CO heifers (average of 0.61 vs. 0.49 kg/day, respectively).

The apparent digestibility coefficients of the CP were greater (*p* < 0.05) in supplemented heifers compared to the control heifers (average of 0.404 vs. −0.037 g/g, respectively) during the experimental period (Table 3). In contrast, supplementation did not affect (*p* > 0.10) the apparent digestibility coefficients of the OM, apNDF, or DOM. In the middle third of gestation, frequency or supplementation plan did not influence (*p* > 0.10) the apparent digestibility coefficients of the OM, CP, apNDF, or DOM. In the same way, there was no effect (*p* > 0.10) of the frequency or supplementation plan on the digestibility coefficients of the OM, CP, apNDF, or DOM, observing only a trend (*p* = 0.085) towards increased CP digestibility in IN heifers’ compared to CO heifers (0.551 vs. 0.448 g/g) in the last third of gestation.

### 3.2. Productive Response

The ADG in prepartum showed higher values in the middle third of gestation compared to the last third of gestation (average of 0.382 vs. 0.120 kg/day, respectively; *p* < 0.001). Thus, a greater BWec (average of 464.8 vs. 439 kg; *p* = 0.008) and ADG (average of 0.268 vs. 0.140 kg/day; *p* < 0.001) was observed, as well as a trend towards an increased BCS (5.9 vs. 5.3; *p* = 0.052) and REA (44.9 vs. 39.1 cm^2^; *p* = 0.099) in supplemented heifers compared to control heifers in the prepartum period (Table 4). However, the supplementation did not affect (*p* > 0.10) the STF in the prepartum period, BW at 60 days, ADG, REA, or STF of beef heifers in the postpartum period.

There was an effect (*p* = 0.020) of the supplementation plan on STF, which was greater in IN heifers compared to CO heifers in the prepartum period (average of 2.92 vs. 2.23 mm, respectively; Table 4). Nonetheless, BWec, BW, ADG, BCS, REA, and STF were not affected by frequency or supplementation plan during the prepartum and postpartum periods. Likewise, the performance of the calves was not affected (*p* > 0.10) by supplementation, frequency, or supplementation plan in the prepartum and postpartum periods.

The average milk yield, milk_4%_, fat, protein, lactose, and total solids were not affected (*p* > 0.10) by the treatments (Table 5). However, there was an effect (*p* < 0.001) of the collection day on milk protein concentration, which was greater at 35 days compared to 50 days postpartum (average of 4.28 vs. 3.06%, respectively). In contrast, milk yield (average of 5.07 vs. 5.13 kg/day, respectively), milk_4%_ (average of 5.52 vs. 5.33 kg/day, respectively), lactose (average of 6.61 vs. 4.67%, respectively), fat (average of 4.57 vs. 4.28%, respectively), and total solids milk concentration (average of 13.31 vs. 13.10%, respectively) were not affected (*p* > 0.10) by collection days.

### 3.3. Metabolite and Hormone Concentration

**Figure 1:**
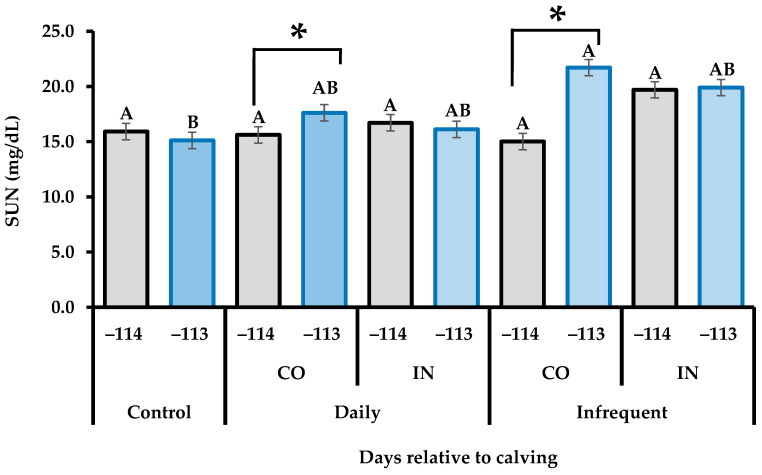
Serum urea nitrogen (SUN) concentrations of super-precocious beef heifers submitted to different feeding supplementation during the middle third of gestation: −114: day relative to calving when both daily heifers and infrequent heifers were supplemented; −113: day relative to calving on which infrequent heifers did not receive a supplement. Control: unsupplemented heifers; Daily: daily supplementation (seven times per week); Infrequent: infrequent supplementation (three times per week; Monday, Wednesday, Friday); CO: constant amount of supplement (1 kg/day in both the middle and last third of gestation); IN: increasing amount of supplement (0.5 and 1.5 kg/day in the middle and last third of gestation, respectively). Means of treatments × days without a common capital letter differ significantly (*p* ≤ 0.05). Days with asterisks (*) are significantly different from each other (*p* < 0.05).

There was an interaction effect (*p* < 0.001) between treatments and collection days to SUN in the middle third of gestation (Table 6). The study of this effect demonstrated that SUN was greater (*p* = 0.042) at −113 days relative to calving in the Infrequent-CO heifers compared to control heifers, and it was also greater at −113 days relative to calving compared to −114 postpartum days in both Daily-CO (*p* = 0.005) and Infrequent-CO (*p* < 0.010) heifers (Figure 1). However, no effects (*p* > 0.10) of supplementation, frequency, or supplementation plan were observed on blood concentrations of total proteins, albumin, glucose, IGF-1, NEFA, and βHB.

**Figure 2:**
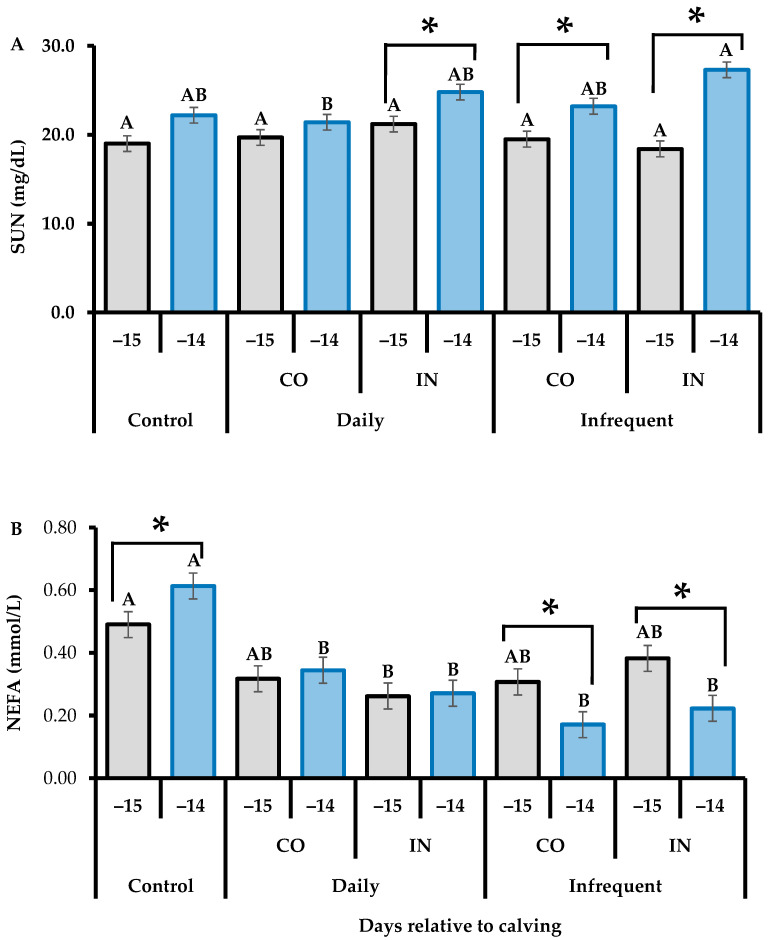
Serum urea nitrogen (SUN) (**A**) and blood non-esterified fatty acids (NEFA) (**B**); concentration of super-precocious beef heifers submitted to different feeding supplementation during the last third of gestation. Control: unsupplemented heifers; Daily: daily supplemented (seven times per week); Infrequent: infrequent supplementation (three times per week; Monday, Wednesday, Friday); CO: constant amount of supplement (1 kg/day in both the middle and last third of gestation); IN: increasing amount of supplement (0.5 and 1.5 kg/day in the middle and last third of gestation, respectively). Means of treatments × days without a common capital letter differ significantly (*p* ≤ 0.05). Days with asterisks (*) are significantly different from each other (*p* < 0.05).

A significant interaction effect of treatment × collection day was observed for SUN (*p* = 0.016) and NEFA (*p* = 0.024) in the last third of gestation (Table 6). A closer examination of this effect evidenced that Infrequent-IN heifers were greater (*p* = 0.024) than Daily-CO heifers at −14 days relative to calving. Additionally, Daily-IN (*p* = 0.006), Infrequent-IN (*p* < 0.001), and Infrequent-CO (*p* < 0.010) heifers all showed greater levels of SUN at −14 days relative to calving compared to −15 days relative to calving (Figure 2A). Evaluation of the interaction for NEFA concentration indicated that control heifers showed greater levels (*p* < 0.05) at −15 and −14 days relative to calving compared to supplemented heifers, and control heifers also had greater (*p* = 0.010) blood NEFA concentrations at −14 days relative to calving compared to −15 days relative to calving (Figure 2B). Infrequent heifers showed lower (*p* < 0.05) levels of NEFA at −14 days relative to calving than at −15 days relative to calving. Nevertheless, there was no effect (*p* > 0.10) of treatments on blood total proteins, albumin, glucose, and βHB concentrations (Table 6).

In the postpartum period, the frequency × supplementation plan interaction for blood albumin concentration (*p* = 0.010) showed that Infrequent-IN heifers had greater levels (Table 6). However, supplemented heifers during gestation had lower albumin (3.10 vs. 3.35 g/dL; *p* < 0.001) and greater NEFA (0.093 vs. 0.049 mmol/L; *p* = 0.045) concentrations compared to control heifers. Supplementation plans alter (*p* = 0.003) blood NEFA concentrations in the postpartum period, being greater in IN heifers relative to CO heifers (0.126 vs. 0.061 mmol/L).

**Figure 3:**
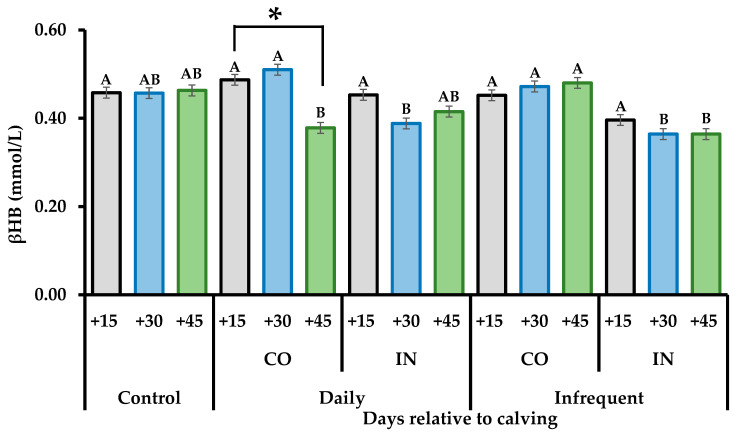
Blood beta hydroxybutyrate (βHB) concentration of super-precocious beef heifers submitted to different feeding supplementation during gestation. Control: unsupplemented animals; Daily: daily supplemented; Infrequent: infrequent supplementation (Monday, Wednesday, Friday); CO: constant amount of supplement (1 kg/day in both the middle and last third of gestation); IN: increasing amount of supplement (0.5 and 1.5 kg/day in the middle and last third of gestation, respectively). Means of treatments × days without a common capital letter differ significantly (*p* ≤ 0.05). Days with asterisks (*) are significantly different from each other (*p* < 0.05).

A trend towards an interaction (*p* = 0.066) for treatment × collection day for βHB concentration was observed (Table 6). Evaluation of this study demonstrated that CO heifers had greater levels at 30 days postpartum compared to IN heifers. Additionally, Infrequent-CO heifers showed greater βHB concentrations at 45 days postpartum compared to Daily-CO and Infrequent-IN heifers. Lower levels (*p* < 0.004) of βHB at 45 days postpartum compared to 15 or 30 postpartum days in Daily-CO heifers were observed (Figure 3). Nonetheless, there was no effect (*p* > 0.10) of treatment on SUN, total proteins, glucose, IGF-1, and progesterone concentrations.

## 4. Discussion

Studies with cattle on grazing indicate that protein supplementation can substantially increase DM intake as well as performance [28]. In our study, this pattern was observed to reinforce the additive effect of supplementation. Additionally, protein supplementation can increase CP content to nearly 100 g CP/kg DM, optimizing forage intake [29]. In this experiment, supplementation (daily or infrequent) increased dietary CP content to 12.8%. It has been pointed out that the adequacy of the dietary protein-to-energy ratio is one of the main indicators of the intake patterns of cattle-fed tropical forages. Maximum forage intake has been observed with a dietary CP/DOM ratio of 210 g/kg [30]. The dietary CP/DOM ratio for unsupplemented (control) and supplemented heifers was, on average, 139 and 232 g/kg, respectively.

Despite the occurrence of previously mentioned elements—such as low forage quality—that could favor greater forage DM intake by supplemented animals, this behavior did not occur. However, a tendency to increase in apNDF and iNDF intake was observed with daily and infrequent supplementation. Based on these responses, two situations can be considered. First, when supplements rich in both nitrogen compounds and easily digestible carbohydrates are included, similar levels of grass intake can be observed. This occurs because the increase in voluntary intake caused by nitrogen compounds is counterbalanced by the reduction in intake caused by energy compounds [31]. Second, during the last third of gestation, exponential fetal growth occurs, close to parturition of the dams, which further imposes constraints on rumen capacity and intensely decreases their feed intake [32,33,34]. In our study, we observed an average reduction of 17.8% in forage intake from the middle to the last third of gestation.

Daily and infrequent supplementation increased CP and OM intake during the gestation due to the additional supply of protein and organic matter provided by the supplement. Heifers that were supplemented daily or infrequently in the gestation had a greater CP digestibility than unsupplemented heifers. Likewise, IN heifers, which received a greater amount of supplement in the last third of gestation (IN heifers), had greater CP digestibility. Such a pattern has been associated with the supplementation of the animals and its positive effect on the degradation of this component in the diet. Protein or protein-energetic concentrates usually have a higher digestibility than forage [35]. The greater CP and OM intake resulted in a higher DOM intake for daily and infrequent heifers.

As we hypothesized, productive and metabolic performance were improved by supplementation and were not negatively influenced by the frequency of supplementation. However, the supplementation plans offered during gestation did not affect productive performance during postpartum.

The tendency towards increases in intake of total DM and increases in CP, OM, and DOM intake in supplemented heifers during gestation increased ADG, BWec, and BCS at calving. The REA is positively correlated with the muscularity of the animals [36]. Thus, as well as ADG and BWec, the tendency towards the larger REA of heifers supplemented daily and infrequently indicates more significant growth, reflecting the greater intake by these animals. Supporting this reasoning, primiparous cows seem more sensitive to nutrient intake, and consequently, BCS changes are more prominent than in non-primiparous cows [25].

The BWec of heifers in the prepartum period showed an average differential gain in supplemented animals compared to the unsupplemented ones, by an average of 25.8 kg. These results show the positive effects of daily and infrequent protein supplementation during prepartum for grazing beef heifers.

On the other hand, the greater STF of IN heifers during prepartum indicates a higher gain in subcutaneous fat, suggesting an increase in adipose tissue deposition, which can be attributed to the greater intake of CP close to parturition because of a greater nutritional plane. The above corroborates the positive effects of protein supplementation (daily or infrequent), with increasing amounts of supplementation during gestation to coincide with nutrient demands increasing due to the accelerated growth of the fetus [9,10].

Overall, according to Cappelloza et al. [6], the reduction of the protein supplement amount does not negatively affect pasture DMI and nitrogen status and the recommended protein supplementation levels ≤ 0.6 g/kg of BW are to maintain acceptable levels of intake and digestibility of nutrients while reducing supplementation costs, in addition to increasing productive performance during prepartum and postpartum periods.

Supplementation during the last third of gestation has been reported as an important factor promoting an increase in fetal growth, altering calf birth weight [25]. However, this effect was not observed in this study. Despite improving the productive performance of heifers at calving, supplementation daily or infrequently did not increase calf birth weight and calf performance or change milk yield. The good BCS during gestation and calving of all heifers did not allow for an effect of the treatments on the BW of the calves. In addition, the good quality of the forage in the postpartum period (106 g CP/kg DM) probably enabled the dams to express their maximum milk yield potential without altering their offspring’s performance.

The higher concentration of SUN only on the second collection day (the day when only daily heifers received supplement) in animals supplemented daily or infrequently independent of the supplementation plan, indicates not only the increase in transfer of ammonia from the rumen to blood but also the capacity to recycle nitrogen and adjust its excretion. This was evidenced mainly in infrequent heifers, which showed an average differential SUN of 3.95 and 5.70 mg/dL compared to daily heifers and control heifers, respectively. It may also suggest a greater mobilization of muscle tissue from unsupplemented heifers, promoting the conceptus’ weight gain. This pattern may have contributed to increased serum nitrogen concentration [37], not resulting in a greater SUN concentration in animals that consumed more CP.

The similar blood total proteins concentrations between treatments during the experiment and albumin concentrations in the prepartum indicate that the animals were in a similar protein status despite the low quality of the pasture and supplementation during gestation. Presumably, this is also an effect of the ruminant ability to adjust the use and excretion of nitrogen, as mentioned previously. However, heifers supplemented during gestation may have had a more intense energy balance in the postpartum period, which can be inferred from their larger maintenance requirement (due to greater BW) and greater NEFA concentrations. In this sense, animals under these conditions tend to have lower blood albumin concentrations. This process may be associated with using a greater proportion of endogenous amino acids as gluconeogenesis precursors [38]. Evaluating the metabolic profile of steers, Montanholi et al. [39] observed that more efficient animals had lower serum albumin concentrations. In our study, albumin concentrations were lowest at 15 days postpartum compared to 30 and 45 days, which may reflect the animals’ greater efficiency at the beginning of lactation due to the tendency for more pronounced negative energy balance (NEB) [38].

Although an increase in DM, OM, CP, and DOM intake was observed in daily and infrequent heifers, there was no difference in blood glucose concentrations between the supplemented treatments and the control group. This may be because glucose is a less expressive indicator to assess energy status due to the insensitivity of glycemia to moderate nutritional changes. Furthermore, glucose is a stress-sensitive metabolite [40], which may have caused the difference in concentration only between collection days in the middle third of gestation, as minor variations may occur in the handling of the animals to the corral and collection.

Greater energy intake and consequent improvements in energy balance are associated with increased circulating IGF-1 [5,41]. This fact corroborates the higher concentrations of IGF-1 in daily and infrequent heifers in the last third of gestation, as they showed higher intake, thus improving energy balance. Although there was also greater consumption during the middle third of gestation, there was no effect of supplementation on IGF-1 concentrations. This is likely associated with a less challenging energy balance at this stage, confirmed by the lower energy demand of gestation and higher absolute values of IGF-1 and ADG. Similar concentrations of IGF-1 in the postpartum period are expected due to the concentration of anabolic hormones being more dependent on nutritional changes than body condition [42]. All dams were maintained in similar nutritional conditions in this period, indicating no nutritional variation between treatments.

The NEB positively correlates with serum NEFA concentration [38,41]. During gestation, unsupplemented heifers showed a greater NEB, which can be inferred from lower intake rates. This was reflected in lower ADG, which is consistent with the higher concentrations of NEFA presented at the end of gestation by unsupplemented animals. According to Ndlovu et al. [43] and Pogliane et al. [44], increases in NEFA values are due to increased mobilization of body fat reserves, so body reserves at birth can also influence postpartum NEFA concentrations. Vizcarra et al. [4] observed a quadratic effect of BCS at birth on NEFA concentration, reiterating the reflection of body condition at birth in the postpartum period. In animals with lower BCS and SFT at birth in our study, lower postpartum NEFA concentration was possibly a reflection of lower fat mobilization, due to its reduced availability in the body of these animals. The lower BWec at calving of unsupplemented heifers may also have contributed to their lower postpartum NEFA concentrations, as they have lower maintenance requirements, tending to have better NEB compared to animals with greater BWec kept in a similar nutritional condition.

Many blood analytes vary throughout the day, and variations occur mainly during the day [45], which can be explained by greater intake activity during the night [46]. In our study, although all collections were performed at the same time, the animals’ grazing habits over the days may vary depending on external factors, which may affect the concentrations of blood analytes collected on the following days. Thus, differences between collection days in blood concentrations of SUN, total proteins, glucose, IGF-1, and βHB in the middle third of gestation and in blood SUN and NEFA in the last third of gestation may be (at least in part) a consequence of daily variation in grazing habits.

According to Lalman et al. [47], a longer service period is typically expected when cows have lower BCS at the beginning of lactation. However, on average, animals from all treatments presented satisfactory BCS to obtain optimized reproductive performance [48], and it is possible that the difference in BCS presented is not sufficient to influence reproductive efficiency [49]. These facts corroborate the lack of difference in progesterone concentrations observed in our study (average of 2.29 ng/mL).

Similar nutritional and metabolic performance between animals that were supplemented daily or infrequently can be explained by the nitrogen recycling capacity of ruminants [50]. This can significantly contribute to the supply of ruminal nitrogen [51], maintaining it as relatively constant. Consequently, the frequency of supplementation did not influence productive performance at the end of gestation and in the postpartum period.

## 5. Conclusions

Protein supplementation during gestation improves the prepartum productive performance of beef heifers on pasture. The frequency or supplementation plans offered in the prepartum period do not negatively impact their performance or metabolic responses. Therefore, a decrease in the frequency of protein supplementation to three times per week and providing 0.5 and 1.5 kg/day of supplement during the middle and last third of gestation, respectively, is recommended.

## Figures and Tables

**Table 1:** Chemical composition of the supplement and forage during the experimental period.

Item	Supplement	Forage ^2^	Forage ^3^	Forage ^4^	Forage ^5^	Forage ^6^
Dry matter (as-fed)	878.00	363.00 ± 0.039	592.00 ± 0.057	407.00 ± 0.018	580.00 ± 0.048	328.00 ± 0.052
Organic matter (g/kg DM)	953.00	935.00 ± 1.824	939.00 ± 2.012	934.00 ± 1.935	935.00 ± 2.142	923.00 ± 1.842
Crude protein (g/kg DM)	250.00	58.00 ± 1.058	51.00 ± 0.317	61.00 ± 0.357	60.00 ± 0.947	106.00 ± 0.402
apNDF ^1^ (g/kg DM)	351.00	698.00 ± 4.563	694.00 ± 3.367	679.00 ± 4.067	677.00 ± 4.217	557.00 ± 4.795
Indigestible NDF (g/kg DM)	80.00	255.00 ± 1.065	273.00 ±4.061	296.00 ± 3.864	296.00 ± 4.362	173.00 ± 1.383

^1^ apNDF: neutral detergent fiber corrected for ash and protein residue. ^2^ Samples obtained via hand plucking in the digestibility trial during the middle third of gestation. ^3^ Samples obtained via hand plucking in the digestibility trial during the last third of gestation. ^4,5,6^ Average values of samples obtained via hand plucking in the middle third of gestation, last third of gestation, and postpartum period, respectively.

**Table 2:** Effects of supplementation plans and frequency on voluntary intake of grazing super-precocious pregnant beef heifers.

Item ^1^	Treatments ^2^					SEM	*p*-Value ^3^			
	Control	Daily		Infrequent			C vs. S	P	F	P × F
		CO	IN	CO	IN					
Intake in the middle third of gestation										
Total DM (kg/day)	4.62	6.62	5.99	7.72	5.97	0.757	0.062	0.177	0.510	0.493
Forage DM (kg/day)	4.62	5.62	5.50	6.71	5.47	0.757	0.200	0.406	0.510	0.493
Organic matter (kg/day)	4.08	6.23	5.58	7.20	5.62	0.646	0.032	0.142	0.466	0.500
Crude protein (kg/day)	0.22	0.52	0.46	0.74	0.44	0.084	0.017	0.083	0.307	0.211
apNDF (kg/day)	3.10	4.35	3.98	4.68	4.07	0.441	0.059	0.308	0.656	0.796
Indigestible NDF (kg/day)	1.19	1.53	1.54	1.53	1.40	0.145	0.095	0.675	0.664	0.637
DOM (kg/day)	1.84	2.64	2.29	3.75	2.50	0.556	0.180	0.209	0.291	0.450
CP/DOM (kg/day)	130.0	207.0	219.0	216.0	180.0	38.900	0.141	0.768	0.723	0.568
Intake in the last third of gestation										
Total DM (kg/day)	4.47	5.74	5.92	5.67	6.14	0.277	0.004	0.283	0.806	0.615
Forage DM (kg/day)	4.47	4.74	4.42	4.67	4.64	0.277	0.634	0.551	0.806	0.615
Organic matter (kg/day)	4.18	5.41	5.56	5.35	5.78	0.260	0.004	0.303	0.757	0.601
Crude protein (kg/day)	0.22	0.48	0.58	0.49	0.64	0.023	<0.001	0.002	0.127	0.306
apNDF (kg/day)	3.09	3.62	3.59	3.59	3.75	0.196	0.045	0.756	0.726	0.630
Indigestible NDF (kg/day)	1.22	1.48	1.37	1.32	1.39	0.103	0.184	0.872	0.540	0.457
DOM (kg/day)	1.56	2.24	2.34	2.33	2.40	0.235	0.031	0.745	0.761	0.954
CP/DOM (kg/day)	139	209	243	216	260	21.5	0.012	0.130	0.621	0.825

^1^ DM: dry matter; apNDF: neutral detergent fiber corrected for ash and protein residue; DOM: digested organic matter. ^2^ Control: unsupplemented animals; Daily: daily supplemented; Infrequent: infrequent supplementation (Monday, Wednesday, Friday); CO: constant amount of supplement (1 kg/day in both the middle and last third of gestation); IN: increasing amount of supplement (0.5 and 1.5 kg/day in the middle and last third of gestation, respectively). ^3^ C vs. S: control versus supplemented; P: effect of the supplementation plan; F: effect of the supplementation frequency; P × F: effect of the interaction between supplementation plan and supplementation frequency.

**Table 3:** Effects of supplementation plans and frequency on apparent digestibility coefficients of grazing super-precocious pregnant beef heifers.

Item ^1^	Treatments ^2^					SEM	*p*-Value ^3^			
	Control	Daily		Infrequent			C vs. S	P	F	P × F
		CO	IN	CO	IN					
Digestibility coefficients in the middle third of gestation										
Organic matter (g/g)	0.412	0.423	0.402	0.497	0.440	0.045	0.598	0.429	0.267	0.709
Crude protein (g/g)	−0.037	0.354	0.358	0.545	0.357	0.069	0.002	0.240	0.227	0.220
apNDF (g/g)	0.548	0.504	0.483	0.532	0.538	0.039	0.468	0.861	0.327	0.745
DOM (g/kg DM)	387	398	375	464	414	42.9	0.614	0.435	0.273	0.764
Digestibility coefficients in the last third of gestation										
Organic matter (g/g)	0.372	0.409	0.423	0.435	0.444	0.032	0.170	0.719	0.491	0.946
Crude protein (g/g)	0.030	0.432	0.511	0.463	0.591	0.049	<0.001	0.085	0.300	0.625
apNDF (g/g)	0.495	0.047	0.478	0.511	0.496	0.032	0.865	0.933	0.398	0.739
DOM (g/kg DM)	349	394	407	410	419	35.8	0.221	0.917	0.898	0.747

^1^ DM: dry matter; apNDF: neutral detergent fiber corrected for ash and protein residue; DOM: digested organic matter; CP/DOM: crude protein and DOM ratio. ^2^ Control: unsupplemented animals; Daily: daily supplemented; Infrequent: infrequent supplementation (Monday, Wednesday, Friday); CO: constant amount of supplement (1 kg/day in both the middle and last third of gestation); IN: increasing amount of supplement (0.5 and 1.5 kg/day in the middle and last third of gestation, respectively). ^3^ C vs. S: control versus supplemented; P: effect of the supplementation plan; F: effect of supplementation frequency; P × F: effect of the interaction between supplementation plan and supplementation frequency.

**Table 4:** Effects of supplementation plans and frequency on productive performance of grazing super-precocious beef heifers during prepartum and postpartum periods.

Item ^1^	Treatments ^2^					SEM	*p*-Value ^3^			
	Control	Daily		Infrequent			C vs. S	P	F	P × F
		CO	IN	CO	IN					
Initial BW (kg)	405	404	408	410	406	21.6	0.927	0.996	0.944	0.860
Prepartum										
BWec (kg)	439	469	462	461	467	5.4	0.008	0.970	0.777	0.289
ADG (kg/day)	0.140	0.301	0.266	0.266	0.239	0.0217	<0.001	0.702	0.695	0.257
BCS	5.3	6.0	5.7	5.8	6.0	0.21	0.052	0.700	0.904	0.292
REA (cm^2^)	39.1	47.1	45.8	40.6	46.2	2.70	0.099	0.396	0.241	0.210
STF (mm)	2.84	2.26	3.22	2.19	2.61	0.225	0.282	0.020	0.151	0.255
Postpartum										
BW at 60 d (kg)	402	426	428	417	425	13.7	0.207	0.741	0.686	0.830
ADG (kg/day)	0.385	0.217	0.013	0.236	0.363	0.2270	0.510	0.871	0.451	0.496
BCS	5.0	5.7	5.1	4.9	5.3	0.26	0.346	0.746	0.236	0.133
REA (cm^2^)	41.1	42.5	41.9	41.1	42.2	2.47	0.756	0.922	0.823	0.747
STF (mm)	1.85	2.07	2.40	1.67	2.06	0.199	0.339	0.141	0.104	0.856
Offspring										
BW at birth (kg)	32.9	33.0	31.1	29.9	32.3	1.90	0.544	0.875	0.630	0.291
BW at 60 d (kg)	71.0	69.4	71.3	68.3	69.1	3.58	0.733	0.724	0.680	0.892
ADG (kg/day)	0.700	0.675	0.741	0.697	0.674	0.0683	0.965	0.774	0.773	0.557

^1^ BW: body weight; BWec: estimated body weight at calving; ADG: average daily gain; BCS: body condition score; REA: ribeye area; STF: subcutaneous thickness fat. ^2^ Control: unsupplemented animals; Daily: daily supplemented; Infrequent: infrequent supplementation (Monday, Wednesday, Friday); CO: constant amount of supplement (1 kg/day in both the middle and last third of gestation); IN: increasing amount of supplement (0.5 and 1.5 kg/day in the middle and last third of gestation, respectively). ^3^ C vs. S: control versus supplemented; P: effect of the supplementation plan; F: effect of supplementation frequency; P × F: effect of the interaction between supplementation plan and supplementation frequency.

**Table 5:** Effects of supplementation plans and frequency on milk yield and composition of grazing super-precocious beef heifers.

Item ^1^	Treatments ^2^					SEM	*p*-Value ^3^					
	Control	Daily		Infrequent			C vs. S	P	F	P × F	D	T × D
		CO	IN	CO	IN							
Milk yield (kg/day)	4.84	4.85	5.41	5.27	5.15	0.645	0.658	0.751	0.909	0.622	0.592	0.499
Milk_4%_ (kg/day)	5.05	5.36	5.69	5.46	5.62	0.762	0.593	0.783	0.958	0.941	0.327	0.866
Fat (%)	4.46	4.64	4.25	4.16	4.62	0.331	0.905	0.925	0.888	0.235	0.169	0.976
Protein (%)	3.64	3.88	3.50	3.48	3.48	0.300	0.899	0.999	0.906	0.269	<0.001	0.923
Lactose (%)	4.59	4.59	4.79	4.61	4.64	0.142	0.691	0.449	0.691	0.584	0.383	0.854
Total solids (%)	13.20	13.50	13.10	12.80	13.50	0.420	0.997	0.744	0.756	0.253	0.246	0.819

^1^ Milk_4%_: milk yield corrected to 4% of fat. ^2^ Control: unsupplemented animals; Daily: daily supplemented; Infrequent: infrequent supplementation (Monday, Wednesday, Friday); CO: constant amount of supplement (1 kg/day in both the middle and last third of gestation); IN: increasing amount of supplement (0.5 and 1.5 kg/day in the middle and last third of gestation, respectively). ^3^ C vs. S: control versus supplemented; P: effect of the supplementation plan; F: effect of supplementation frequency; P × F: effect of the interaction between supplementation plan and supplementation frequency; D: effect of the collection day; T × D: effect of the interaction between treatment and collection day.

**Table 6:** Effects of supplementation plans and frequency on metabolic responses of grazing super-precocious beef heifers during prepartum and postpartum periods.

Item ^1^	Treatments ^2^					SEM	*p*-Value ^3^					
	Control	Daily		Infrequent			C vs. S	P	F	P × F	D	T × D
		CO	IN	CO	IN							
Middle third of gestation												
SUN (mg/dL)	15.5	16.6	16.4	18.4	19.8	1.74	0.289	0.197	0.726	0.663	<0.001	<0.001
Total protein (g/dL)	6.86	6.55	6.83	6.73	6.95	0.145	0.530	0.282	0.120	0.893	0.009	0.109
Albumin (g/dL)	3.38	3.30	3.33	3.35	3.46	0.071	0.785	0.217	0.388	0.580	0.390	0.103
Glucose (mg/dL)	59.6	60.4	55.5	60.1	59.0	1.78	0.709	0.418	0.148	0.362	0.050	0.130
IGF-1 (ng/mL)	325	302	321	362	302	48.9	0.954	0.692	0.692	0.459	<0.001	0.153
NEFA (mmol/L)	0.146	0.153	0.178	0.087	0.208	0.0380	0.819	0.660	0.114	0.262	0.177	0.261
βHB (mmol/L)	0.322	0.354	0.372	0.415	0.382	0.0540	0.364	0.535	0.889	0.653	<0.001	0.161
Last third of gestation												
SUN (mg/dL)	20.2	20.5	23.0	21.3	22.9	1.22	0.321	0.190	0.807	0.732	<0.001	0.016
Total protein (g/dL)	6.55	6.56	6.64	6.68	6.83	0.169	0.482	0.487	0.353	0.860	0.242	0.245
Albumin (g/dL)	3.49	3.27	3.29	3.20	3.35	0.111	0.170	0.489	0.946	0.589	0.513	0.476
Glucose (mg/dL)	50.6	53.8	49.9	54.8	55.3	1.91	0.238	0.377	0.134	0.262	0.918	0.874
IGF-1 (ng/mL)	111	152	173	195	164	16.6	0.012	0.753	0.310	0.141	0.782	0.719
NEFA (mmol/L)	0.551	0.330	0.266	0.239	0.303	0.0560	0.007	0.998	0.615	0.215	0.331	0.024
βHB (mmol/L)	0.651	0.588	0.485	0.485	0.480	0.6460	0.135	0.446	0.436	0.473	0.510	0.114
Postpartum period												
SUN (mg/dL)	16.3	16.3	17.2	17.8	16.1	1.29	0.712	0.759	0.907	0.349	0.118	0.376
Total protein (g/dL)	6.64	6.37	6.54	6.58	6.92	0.212	0.880	0.276	0.216	0.713	0.783	0.430
Albumin (g/dL)	3.35	3.11	3.13	2.91	3.25	0.545	<0.001	0.004	0.500	0.010	0.091	0.673
Glucose (mg/dL)	54.1	55.5	54.5	55.0	55.4	1.73	0.594	0.861	0.899	0.650	0.093	0.508
IGF-1 (ng/mL)	256	257	218	244	231	37.5	0.669	0.497	0.994	0.731	0.844	0.710
NEFA (mmol/L)	0.049	0.052	0.109	0.069	0.142	0.0180	0.045	0.003	0.221	0.712	0.608	0.572
βHB (mmol/L)	0.459	0.458	0.419	0.468	0.375	0.0370	0.490	0.105	0.665	0.498	0.356	0.066
Progesterone (ng/mL)	1.10	2.94	5.63	1.37	0.41	2.18	0.574	0.711	0.164	0.444	0.247	0.500

^1^ SUN: serum urea nitrogen; IGF-1: insulin-like growth factor type I; NEFA: non-esterified fatty acids; βHB: beta hydroxybutyrate. ^2^ Control: unsupplemented animals; Daily: daily supplemented; Infrequent: infrequent supplementation (Monday, Wednesday, Friday); CO: constant amount of supplement (1 kg/day in both the middle and last third of gestation); IN: increasing amount of supplement (0.5 and 1.5 kg/day in the middle and last third of gestation, respectively). ^3^ C vs. S: control versus supplemented; P: effect of the supplementation plan; F: effect of supplementation frequency; P × F: effect of the interaction between supplementation plan and supplementation frequency; D: effect of the collection day; T × D: effect of the interaction between treatment and collection day.

## Data Availability

The data presented in this study are available upon request from the corresponding author.
